# An Identification Method for Road Hypnosis Based on Human EEG Data

**DOI:** 10.3390/s24134392

**Published:** 2024-07-06

**Authors:** Bin Wang, Jingheng Wang, Xiaoyuan Wang, Longfei Chen, Han Zhang, Chenyang Jiao, Gang Wang, Kai Feng

**Affiliations:** 1 College of Electromechanical Engineering, Qingdao University of Science and Technology, Qingdao 266000, China; wangbin@mails.qust.edu.cn (B.W.); chenlongfei@mails.qust.edu.cn (L.C.); zhanghan@mails.qust.edu.cn (H.Z.); jiaochenyang@mails.qust.edu.cn (C.J.); wanggang@mails.qust.edu.cn (G.W.); fengkai@mails.qust.edu.cn (K.F.); 2 Department of Mathematics, Ohio State University, Columbus, OH 43220, USA

**Keywords:** road hypnosis, EEG, vehicle, drivers, state identification

## Abstract

The driver in road hypnosis has not only some external characteristics, but also some internal characteristics. External features have obvious manifestations and can be directly observed. Internal features do not have obvious manifestations and cannot be directly observed. They need to be measured with specific instruments. Electroencephalography (EEG), as an internal feature of drivers, is the golden parameter for drivers’ life identification. EEG is of great significance for the identification of road hypnosis. An identification method for road hypnosis based on human EEG data is proposed in this paper. EEG data on drivers in road hypnosis can be collected through vehicle driving experiments and virtual driving experiments. The collected data are preprocessed with the PSD (power spectral density) method, and EEG characteristics are extracted. The neural networks EEGNet, RNN, and LSTM are used to train the road hypnosis identification model. It is shown from the results that the model based on EEGNet has the best performance in terms of identification for road hypnosis, with an accuracy of 93.01%. The effectiveness and accuracy of the identification for road hypnosis are improved in this study. The essential characteristics for road hypnosis are also revealed. This is of great significance for improving the safety level of intelligent vehicles and reducing the number of traffic accidents caused by road hypnosis.

## 1. Introduction

In 1929, a phenomenon known as “sleeping with the eyes open during driving” was proposed in a research report titled “Sleeping with the Eyes Open” [1]. It is noted in the report that under specific circumstances, automobile drivers may fall into the peculiar state of “sleeping with the eyes open”. According to a survey [2], accidents resulting in death caused by vehicle incidents account for about one-third of all traffic-related fatalities in the United States. Among these accidents, 50% are caused by personal factors such as fatigue and distraction. Williams G.W. et al. [3] described a phenomenon similar to hypnosis that occurs in drivers as a result of prolonged driving in a monotonous environment such as a highway. They pointed out that this phenomenon is characterized by amnesia, a dazed state, and slower reaction times. However, drivers could still maintain a normal driving state. They also clarified the differences between this phenomenon and sleep. Williams G.W. et al. [4] described this phenomenon as a state of drowsiness, with distorted thoughts and judgment. Wertheim A. M. [5] proposed the eye movement theory, which suggested that objects within the driver’s visual field move in a predictable pattern relative to the driver, who must maintain prolonged focus on these objects, thereby easily inducing a phenomenon similar to hypnosis. Cerezuela G. P. et al. [6] believed that, in this hypnosis state, drivers were in an unconscious driving mode.

Xiaoyuan Wang defined road hypnosis as an unconscious driving state formed by a combination of external environmental factors and the psychological state of the driver [7]. The onset of road hypnosis is attributed to repetitive and low-frequency stimuli present in highly predictable driving environments. The specific manifestations of road hypnosis include drivers experiencing perceptual paralysis, reduced attention, and decreased vigilance, accompanied by transient states of confusion, amnesia, and delusion. It is a state that can be induced by multiple factors, including endogenous factors (such as the driver’s susceptibility to hypnosis, fatigue, and circadian rhythms) and exogenous factors (such as road geometry, monotony of the driving task, monotony of the driving context, and vehicle enclosure). When drivers emerge from a state of road hypnosis, they typically experience a marked state of alertness. Drivers often do not remember the events that occurred in the state of road hypnosis. However, drivers have a clear memory of the state they just experienced. Although drivers appear to maintain normal driving behavior in this state, their reaction times are significantly slower than in a normal driving state. Based on the definition of road hypnosis, a preliminary attempt was made with features from eye movements, bioelectrical signals, and their integration to establish a model for identifying the state of road hypnosis [7,8]. Initial results are achieved in this study: the state of road hypnosis is successfully identified using features from eye movements, bioelectrical signals, or a combination of both. However, intrinsic characteristics of the road hypnosis state, such as electroencephalographic (EEG) parameters directly related to human brain activity, have not been utilized in identifying the state of road hypnosis.

Electroencephalography (EEG) is a non-invasive diagnostic technique that assesses the electrical activity of neurons in the brain [9]. By attaching an array of electrodes to the surface of the skull, the electrical potential fluctuations of neuronal groups within the brain can be captured and recorded. The recorded electrical potential fluctuations are typically categorized by their frequency characteristics into γ (30–42 Hz), β (13–30 Hz),α (8–13 Hz), θ (4–8 Hz), and δ (0.5–4 Hz) waves, each corresponding to different physiological and cognitive states of the brain. EEG plays a crucial role in research on driver life detection, serving as the gold-standard indicator for identifying vital signs in drivers. A method for directly monitoring electrical activity in the brain is provided by electroencephalography, which can reflect the physiological and cognitive states of the brain in real time. Models for identifying fatigued driving and distracted driving, which exhibit external characteristics similar to road hypnosis, have shown good performance using electroencephalographic (EEG) data. In existing research, the main methods of electroencephalographic (EEG) data analysis include entropy analysis, power spectral density (PSD) analysis, and the integration of EEG with other physiological parameter features. The inherent complexity and irregularity of brain electrical activity can be revealed by the use of entropy feature extraction techniques. The functional states of the brain can be further understood. Papadelis C. et al. [10] compared EEG signals from five minutes prior to the onset of fatigue and from the first five minutes of driving. They observed a significant increase in the relative band ratio (RBR) of α waves and a decrease in θ waves, along with notable increases in Shannon entropy and Kullback–Leibler entropy. These findings demonstrate that EEG can effectively assess changes in brain activity occurring seconds before sleep/drowsiness events during driving. The quantitative measurements of these data can be used as potential drowsiness indicators for the development of future countermeasure devices for driving fatigue. Tran Y. et al. [11] conducted sample entropy and second-order difference plot analysis of EEG signals of fatigue during driving simulations using central tendency measure (CTM). They found that sample entropy and second-order difference plots made the patterns in EEG signals more pronounced. Kar S. et al. [12] conducted a relative quantification of fatigue during driving by processing EEG signals using entropy methods. They found that the patterns of changes in these entropies were distinct at different stages of fatigue. Rodríguez-Sotelo J. et al. [13] used entropy metrics and the q-algorithm as dimensionality reduction methods, and j-means clustering as a classifier to implement two types of automated sleep stage scoring based on EEG channels. Wang F. [14] studied driving fatigue detection based on electroencephalographic (EEG) features. Fine composite multiscale fluctuation dispersion entropy features were extracted from the θ and β rhythm signals in the collected EEG signals. Mu Z. et al. [15] utilized fuzzy entropy for feature extraction and employed an SVM (support vector machine) as a classification tool, discovering that EEG signals from the frontal lobe can be used to detect the fatigue state of drivers. Min J. et al. [16] analyzed a fusion method of multiple entropies (including spectral entropy, approximate entropy, sample entropy, and fuzzy entropy) based on EEG recordings, and assessed multiple channel areas, thereby effectively detecting the fatigue state of drivers.

Power spectral density (PSD) analysis is a method used to quantify the distribution of power across various frequencies in a signal or time series. This analysis allows for an understanding of how the energy of the signal varies with frequency. The main frequency components within the signal can be identified by this analysis method, which effectively analyzes the periodic changes or oscillatory characteristics of the signal. Balasubramanian V. [17] found through virtual driving experiments that the average power of α waves in EEG signals at the 12th minute was significantly higher than that at the 4th minute, indicating that EEG can be used to determine cognitive fatigue in drivers. Borghini G. [18] discovered that during driving fatigue, the power of theta EEG waves increases in the frontal lobe area of the brain, while the power of alpha EEG waves decreases in the parietal area under challenging driving conditions. Radha M. [19] utilized six different EEG methodologies along with various signal processing features, such as time–domain, frequency–domain, and nonlinear characteristic. They found that classification using EEG signals from the frontal lobe yielded the best results, with its spectral linearity features and random forest (RF) outperforming the support vector machine (SVM) in terms of classification effectiveness. Tuncer T. et al. [20] proposed a method for determining fatigue levels by using power spectral density analysis. This method exhibits a high classification accuracy. Wang H. et al. [21] utilized power spectral density analysis to identify mental fatigue. They employed the wavelet packet transform (WPT) method to obtain frequency components in the θ (4–7 Hz), α (8–12 Hz), and β (13–30 Hz) frequency bands, and computed the power spectral density (PSD) corresponding to the selected channels. Zheng Y. [22] proposed a novel method combining integrated empirical mode decomposition and PSD to explore new EEG features for driver fatigue detection. Liu X [23] performed pairwise estimations of multiple EEG features, including power spectral density (PSD), functional connectivity (FC), and entropy, and used them as inputs for fatigue classification. It was found that the PSD of different frequency bands in the cerebral cortex and the FC between the cerebral cortex and the frontoparietal region have the most significant impact on the cerebral cortex. Huo X.Q. et al. [24] employed a discriminative graph regularized extreme learning machine, integrating EEG with frontal EEG, to detect the fatigue levels of drivers. They discovered that, compared to using only EEG or a frontal electroencephalogram, the integrated model exhibited a higher predictive correlation coefficient and lower RMSE values, enhancing the performance of driver fatigue detection. Gao Z. et al. [25] established a spatiotemporal convolutional neural network based on multichannel EEG signals to detect the fatigue state of drivers, leveraging the temporal and spatial structures of the EEG signals.

Although there are currently no studies on the analysis or identification of drivers’ road hypnosis using EEG data, there are already numerous studies in the medical field that have used EEG data to analyze and assess the hypnosis of humans in non-driving conditions. Anoushiravan Z. [26] compared the effects of hypnosis alone and hypnosis with post-hypnotic suggestions on the Stroop effect and its facilitative and inhibitory components. The mechanisms of hypnosis at the neural level were investigated through the analysis of EEG frequencies. EEG recordings from the Stroop task revealed that participants under the influence of hypnosis exhibited significant increases in θ and β energy in their frontal lobes. Golnaz B. [27] used different EEG features to differentiate levels of hypnosis. These features were extracted from EEG signals recorded during hypnosis from 32 subjects. Golnaz B. proposed a method to assess levels of hypnosis from EEG signals. It replaces traditional subjective clinical tests. Dario Madeo et al. [28] characterized the EEG dynamics of subjects with high and low hypnotizability using a nonlinear method of recursive quantification analysis. Single-channel EEG was briefly recorded by this method using standard mathematical analysis techniques. Aboalayon K. [29] proposed an automatic method for classifying sleep stages based on single-channel EEG. A key feature of this method is the selection of filtering techniques that decompose 10-s EEG signals into different sub-bands, developing new time–domain features for classification with reduced computational time. This method breaks down the EEG signals into five frequency bands: δ, θ, α, β, and γ waves. Alejandro L. [30] assessed the EEG brain activity of participants with high or low hypnotizability scores to understand the levels of hypnotizability reflected in these EEG activities. Dhritiman C. [31] analyzed the spectral entropy of EEG signals to develop indicators for measuring the depth of hypnosis. By correlating the observed results with published entropy algorithms, he described the propagation of cardiac signals in the head and neck area and its impact on EEG data entropy, thus elucidating the mechanisms behind these observations. Subasi et al. [32] demonstrated the effectiveness of wavelet transform in extracting relevant features from EEG signals. Irfan et al. [33] utilized wavelet-based methods for EEG feature extraction with promising results.

In existing research, the intrinsic characteristics of road hypnosis have not been sufficiently studied. Road hypnosis has not been identified with EEG data directly related to human brain activity. A method for identifying road hypnosis in vehicle driving based on the computation of human EEG data is proposed in this study. EEG data are collected from drivers in a state of road hypnosis through vehicle driving experiments and virtual driving experiments. The collected EEG data, after preprocessing, are analyzed using the method of power spectral density analysis. Three types of neural networks—EEGNet, recursive neural networks, and long short-term memory networks—are used to construct models for identifying road hypnosis. The performances of the three models are validated, compared, and analyzed to evaluate their identification capabilities. The validation results indicate that the road hypnosis state model constructed with EEGNet achieves the highest accuracy and demonstrates the best performance in identification.

The contribution of this study as follows:

1. Current research on road hypnosis is relatively sparse, particularly in identifying this state using electroencephalography (EEG), which is a gold-standard parameter. Road hypnosis is systematically examined for the first time in this study with EEG data collected through vehicle driving experiments and virtual driving experiments.
2. Drivers’ physiological and cognitive states can be revealed with EEG signals. In this study, EEG data are preprocessed using power spectral density (PSD) analysis. EEG features are related to road hypnosis to realize the accurate and reliable identification for road hypnosis.
3. The performances of three models—EEGNet, RNN, and LSTM—are compared and analyzed, and the superiority of EEGNet in identifying road hypnosis is demonstrated in this paper. A reliable method for detecting road hypnosis is proposed.

## 2. Experiment Methodology

### 2.1. Experiment Participants

In preliminary exploratory studies [7,8], it was found that some drivers were more prone to experiencing road hypnosis. Therefore, in this study, 45 eligible drivers were recruited as participants through social recruitment. The requirements for the experiment participants were as follows:

1. As novice drivers tend to drive more cautiously, making them less susceptible to road hypnosis, participants selected for the study were drivers with at least 5 years of driving experience, aged between 25 and 60 years, and in good physical and mental health.
2. Drivers with stable occupations, fixed residences, regular commuting routes, and fixed commuting times were selected. Their average daily driving time should not be less than 1 h.
3. Drivers with regular routines, fixed sleep schedules, and good sleep habits were selected.

The basic information about the occupations of drivers in the experimental sample is shown in Table 1.

### 2.2. Experimental Equipment

**Figure 1:**
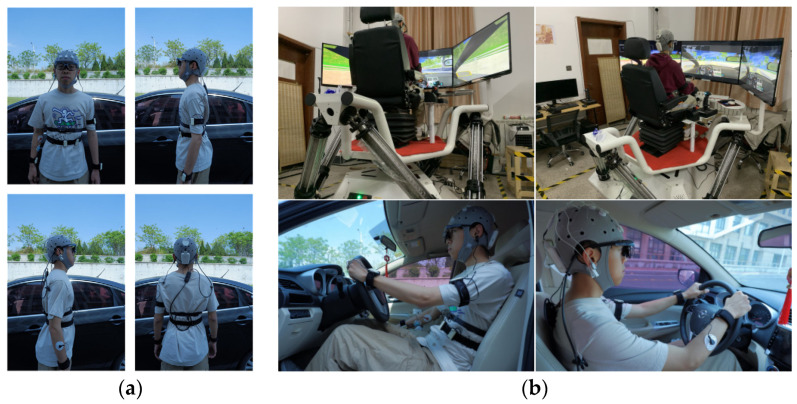
Equipment and environment. (**a**) Photographs of the participants wearing the equipment. (**b**) Environment.

The experimental design of this study consists of two main parts: vehicle driving experiments conducted under real road conditions and virtual driving experiments conducted in controlled environments. The experimental equipment used includes a multifunctional comprehensive experimental test vehicle, a virtual driving simulation platform, EEG equipment, an eye tracker, human factor equipment, laptops, and video recorders. Equipment and environment are shown in Figure 1.

### 2.3. Experimental Procedure

Vehicle driving experiments and virtual driving experiments in this study were conducted in monotonous driving environments such as tunnels or highways. The data for the vehicle experiments were collected in real road environments. The virtual environment in the virtual driving platform was designed to resemble vehicle driving environments, simplifying the experimental process. By conducting this experiment, a large amount of driving data was easily collected, enriching and expanding the experimental data.

#### 2.3.1. Vehicle Driving Experiment

The experimental road for the vehicle driving experiments was selected from the Qingdao Jiaozhou Bay Tunnel and the Jiaozhou Bay Bridge in Qingdao City. Qingdao Jiaozhou Bay Tunnel Road has a total length of 17.9 km, with the tunnel itself being 15.9 km long. The tunnel consists of two separate tubes, each with three lanes, totaling six lanes for both directions. The left tube is 3.875 km long, while the right tube is 3.888 km long. Each tube has a width of 14.8 m and a height of 8 m. Through vehicle driving experiments, it was found that this tunnel is a closed, straight driving section with road lights fixed at regular intervals on both sides of the road. Drivers’ road hypnosis was easily induced. The Jiaozhou Bay Bridge Road has a total length of 42.23 km with a speed limit of 80 km/h. It is a dual six-lane bridge, with a bridge length of 31.63 km. Through vehicle driving experiments, it was found that although the Jiaozhou Bay Bridge is not a closed section, the scenery on both sides of the road consists of extremely monotonous sea views. Drivers’ road hypnosis was easily induced.

In comparison to virtual driving experiments, there are many unstable factors in the process of vehicle driving experiments, such as lane changing and overtaking. To induce a state of road hypnosis in drivers as effectively as possible, the Jiaozhou Bay Tunnel and Jiaozhou Bay Bridge in Qingdao were chosen as the settings for the vehicle driving experiments in this study. The Jiaozhou Bay Tunnel has a total length of 7.797 km, with the sea-crossing section measuring 4.095 km. The road is designed as a six-lane urban arterial road with bidirectional traffic, featuring separated lanes for each direction. The main tunnel cross-section is elliptical. Through vehicle driving experiments, it was found that this tunnel is a closed, straight-line section of road. The tunnel’s road lights are placed at fixed intervals along both sides of the road, which are highly conducive to inducing a state of road hypnosis in drivers. The Jiaozhou Bay Bridge has a total road length of 42.23 km and a speed limit of 80 km/h. This bridge features six lanes with bidirectional traffic and has a bridge length of 31.63 km. Through vehicle driving experiments, it was found that although the Jiaozhou Bay Bridge is not an enclosed section, the driving environment is relatively monotonous. The seascape acts as visual “white noise”, easily inducing a state of highway hypnosis in drivers.

**Figure 2:**
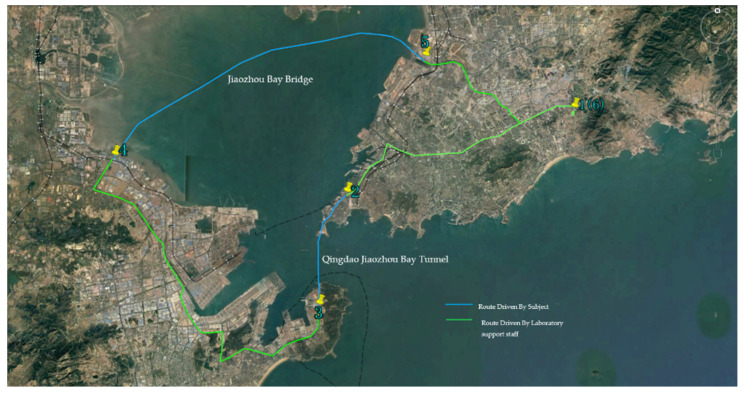
Vehicle driving experiment route.

The experiment times of vehicle driving were selected from 9 a.m. to 12 a.m. In addition to the experimental subjects, there were four laboratory support staff to jointly arrange the experiment. During the experiment, the subjects were required to keep a constant speed of 80 km/h for as long as possible, and drive in a straight line as far as possible to avoid lane changes and overtaking. The specific experimental process was as follows:

(1) Before the start of the experiment, one of the laboratory support staff assisted the subjects in putting on an eye tracker, EEG equipment, human factor equipment, and other instruments and equipment, then recorded the start time and total duration of the experiment during the experiment.
(2) The experiment started at the prescribed time. The driving route for the vehicle driving experiment is shown in Figure 2. One of the laboratory support staff drove the vehicle starting at place 1 and stopped near place 2. The driver was replaced by the subject, and the subject drove the vehicle from place 2 to place 3. During this driving process, one of the laboratory support staff was responsible for observing traffic flow and other road conditions for the co-driver, and reminding the subjects to drive carefully in road sections with large traffic flow and complex driving environments to ensure their safety. One of the laboratory support staff continuously observed the driver’s eye movements and ECG patterns. The time period during which the driver’s eyes were focused ahead and the ECG signal remained stable needed to be recorded by laboratory support staff. At times when the subject might have been alert, an experimental assistant actively asked the driver whether a road hypnosis state had just occurred and recorded it.
(3) After arriving at place 3, laboratory support staff assisted subjects in removing the experimental equipment. Subjects had ten minutes to take a break. A laboratory support staff immediately asked the subjects whether they had experienced fatigue, distraction, or other abnormal driving behaviors during the driving process and recorded them. They observed the experimental videos and the corresponding eye movements and bioelectrical information, then asked the subjects to recall whether they had experienced a road hypnosis state and recorded it.
(4) Then, a laboratory support staff drove the vehicle from place 3 to place 4, and after being replaced as the driver by the subject, the subject continued to drive the vehicle from place 4 to place 5. The experimental process in this period was the same as steps (2) and (3).
(5) After arriving at place 5, the vehicle was driven back to place 1 (point of departure) by a laboratory support staff member. After the completion of a single experiment, the above experimental process was repeated until the data collection of all experimental personnel was complete. After all experiments had been completed, the experimental equipment was sorted out and the experiment was ended.

#### 2.3.2. Virtual Driving Experiment

A straight section of road measuring 50 km in length and 15 km in width, with four lanes for bidirectional traffic, was selected as the setting for the virtual driving experiment. Interference from other vehicles was eliminated during the driving process. Before the experiment, subjects were instructed to ensure they had adequate sleep. The experiment was scheduled to start at 9:00 a.m. and end at 12:30 p.m. In addition to the 45 subjects, three laboratory support staff were involved in arranging the experiment for this study. The specific experimental process was as follows:

(1) Before the start of the experiment, one of the laboratory support staff was responsible for setting up the equipment and assisting the subjects with wearing the necessary devices. During the experiment, this laboratory support staff member was responsible for recording the start time and the total duration of the experiment.
(2) The experiment started at the prescribed time, and subjects were required to maintain a speed of 120 km/h and drive in a single lane for 20 min. One of the laboratory support staff continuously observed the driver’s eye movements and ECG patterns. Time periods when the driver’s eyes were focused ahead and the ECG signal remained stable needed to be recorded by laboratory support staff. At a time when the subject might have been alert, an experimental assistant actively asked the driver whether a road hypnosis state had just occurred and recorded it.
(3) After the completion of each driving session, the laboratory support staff assisted the subjects in removing their experimental equipment. The subjects had ten minutes to take a break After the driving session, one of the laboratory support staff asked the participants if they had experienced fatigue, distraction, or any other unusual driving behaviors during the recent driving process and recorded their responses. They observed the experimental videos and the corresponding eye movement and bioelectrical information, then asked the subjects to recall whether they had experienced a road hypnosis state and recorded it. After this process was complete, the driving experiment was restarted. The experimental procedure remained consistent, and the duration of the experiment was extended to 40 min.

After all experiments had been completed, the experimental equipment was sorted out and the experiment was ended.

## 3. Data Processing and Analysis

### 3.1. Data Preprocessing

#### 3.1.1. Data Screening

**Figure 3:**
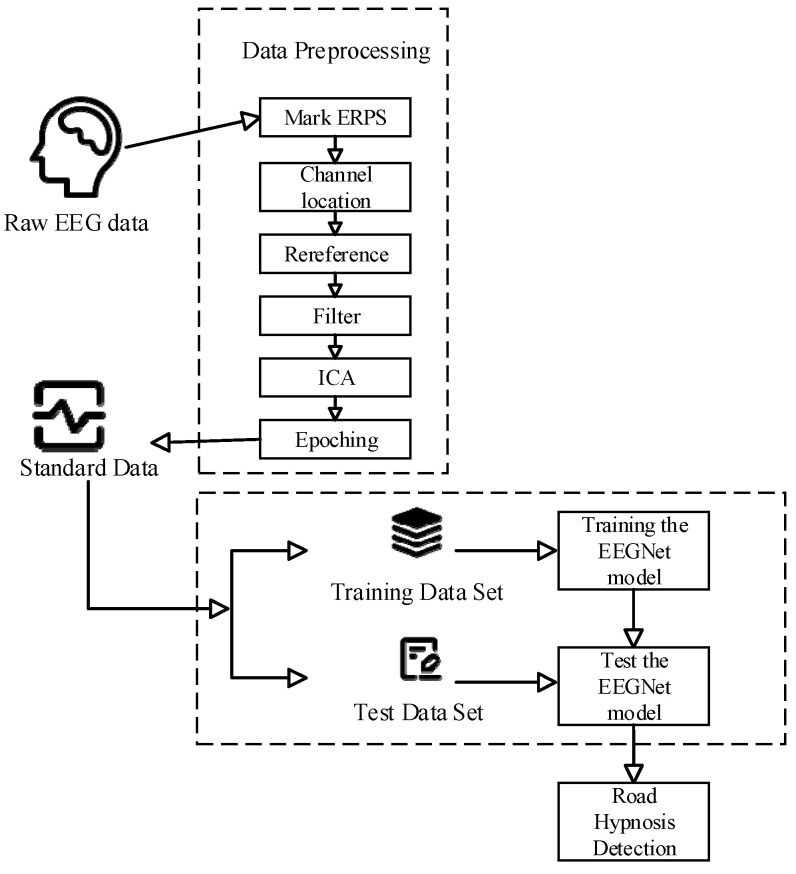
Data processing procedure.

Data processing procedure is shown in Figure 3. A total of 45 sets of data were obtained from the vehicle driving experiments and virtual driving experiments, respectively. The data were analyzed by researchers who had relevant study experience with road hypnosis in our laboratory. They focused on identifying periods during which participants might have experienced road hypnosis using eye movement data and physiological data such as electrocardiograms, electromyograms, and respiratory rates. Whether the participants were in a state of road hypnosis could be determined through this progress. Data segments with specific characteristics of road hypnosis were selected to generate the road hypnosis datasets, which included a vehicle driving experiment dataset and a virtual driving experiment dataset. The data segments where the participants were not in states of road hypnosis were selected to generate normal driving datasets.

After the datasets had been screened, the researchers conducted a manual review of the selected datasets and confirmed their accuracy based on video playback, ultimately assigning final scores to the datasets. During the experiment, some participants exhibited signs of fatigued driving due to factors such as physical exhaustion and improper driving posture. As a result, data from four participants were excluded from the vehicle driving experiments, and data from three participants were excluded from the virtual driving experiments. Ultimately, 41 sets of data from the vehicle driving experiments and 42 sets from the virtual driving experiments were selected for the databases.

#### 3.1.2. Data Annotation

**Figure 4:**
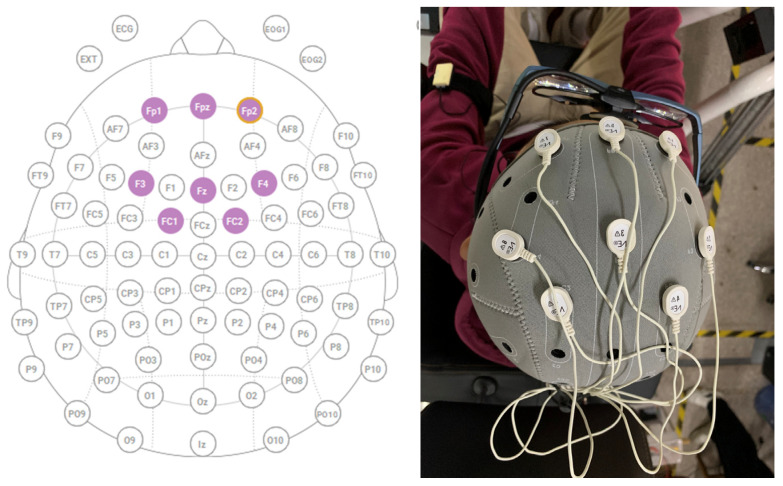
EEG channel distribution diagram.

(1) In this study, the selected EEG channels are located in the frontal lobe area, as shown in Figure 4, including the Fp2, Fpz, Fp1, F4, Fz, F3, FC2, and FC1 channels. The frontal lobe area is central to cognitive functions, attention, and emotional regulation in humans. It is closely associated with executive functions, levels of attention, emotional states, and arousal levels. Road hypnosis involves a decline in a driver’s attention and an increase in reaction time, making the frontal lobe area particularly relevant for studying this phenomenon.

(2) In the technical settings for signal acquisition, a 250 Hz low-pass filter and a 0.1 Hz high-pass filter were used to optimize signal quality and eliminate potential non-EEG noise components that could interfere with the data.

(1) $$H(f)=\frac{1}{1+{\left(\frac{f}{f_{c}}\right)}^{2}}$$

In this case, $f_{c}$ represents the cutoff frequency for the low-pass filter, which is set at 250 Hz.

The high-pass filter was set at 0.1 Hz to remove the DC component and very low-frequency artifacts (such as respiration and motion) from the signal.

(2) $$H(f)=\frac{1}{1+{\left(\frac{f_{c}}{f}\right)}^{2}}$$

In this case, $f_{c}$ represents the cutoff frequency for the high-pass filter, which was set at 0.1 Hz.

(3) Based on the Nyquist sampling theorem, the sampling rate should be at least twice the highest frequency component in the signal. Setting the sampling rate at 500 Hz ensured effective data acquisition while avoiding the burden of excessive data processing associated with higher sampling rates.

(3) $$f_{s}\geq 2f_{\max}$$

In this case, with a sampling rate of 500 Hz, the highest frequency component ($f_{\max}$) in the signal should not exceed 250 Hz.

(4) $$f_{Nyquist}=\frac{f_{s}}{2}$$

The Nyquist frequency is half of the sampling frequency. For a sampling rate of 500 Hz, the Nyquist frequency is indeed 250 Hz. This is the highest frequency that can be unambiguously recorded by the sampling system.

(5) $$f_{s}=k\times 2f_{\max}$$

In this equation, k is the oversampling factor. If k>1, it means that the sampling rate is higher than the minimum requirement of the Nyquist frequency. Oversampling can reduce aliasing and simplify the design of digital filters.

An example of parameter information for the raw EEG data from the 23rd set in the real vehicle driving experiment database is provided in Table 2.

The time periods corresponding to road hypnosis and normal driving in the filtered 41 sets of vehicle driving experiment datasets and 42 sets of virtual driving experiment datasets were labeled as RH (Road Hypnosis) and ND (Normal Driving), respectively, in relation to event-related potentials. In the 41 sets of vehicle driving experiment datasets, there were 6063 event-related potentials classified as Road Hypnosis (RH) and 4183 event-related potentials classified as Normal Driving (ND). In the 42 sets of virtual driving experiment datasets, there were 5182 event-related potentials classified as Road Hypnosis (RH) and 7245 event-related potentials classified as Normal Driving (ND).

#### 3.1.3. Data Processing

(1) Electrode location

The electrode montage was established with a custom XML configuration through mapping each electrode to specific locations on the scalp corresponding to the international 10–20 system. The device consisted of eight primary electroencephalogram (EEG) channels located primarily in the frontal lobe region: Fp2, Fpz, Fp1, F4, Fz, F3, FC2, and FC1.

(2) Re-reference

The reference point for the EEG signals was set to a bilateral mastoid average reference.

The reference electrodes, placed bilaterally at the mastoids and averaged, were positioned further away from the scalp to reduce the influence of volume conduction effects on the EEG signals.

(3) Filtering

Two types of filtering, low-pass and high-pass filtering, were employed in this study. The cutoff frequency of the low-pass filter was set to 30 Hz to focus on the EEG frequency range most closely associated with sleep-related phenomena. The cutoff frequency of the high-pass filter was set to 0.1 Hz, retaining the low-frequency components relevant to hypnosis research while removing extremely low-frequency noise and drift.

To further eliminate artifacts caused by power line interference, a notch filter with a cutoff frequency of 40 Hz was employed in this study. The design of this filter was based on the finite impulse response (FIR) principle. Its output *y*(*n*) was only dependent on the current and a finite number of past input values *x*(*n*), *x*(*n* − 1), …, *x*(*n* − *m*). The relationship between the input and output of an FIR filter was defined according to the following equation:

(6) $$y(n)=\sum_{r=0}^{M}b_{r}x(n-r)$$

**Figure 5:**
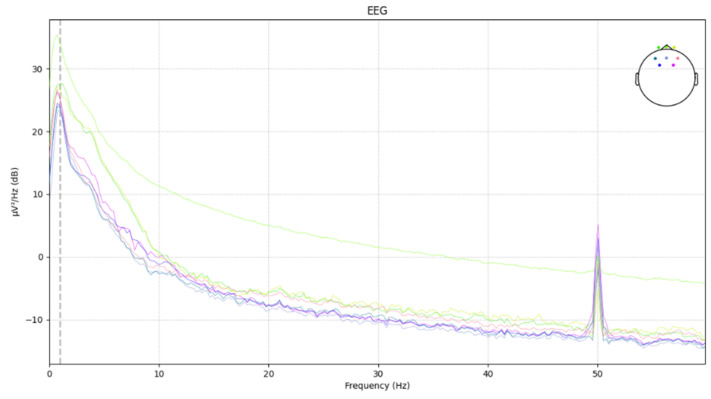
Power spectral density of the EEG after applying low-pass filtering, high-pass filtering, and a notch filter at 40 Hz.

The power spectral density of the EEG after applying low-pass filtering, high-pass filtering, and a notch filter at 40 Hz is shown in Figure 5. 

Based on the methods used in references [32,33], the EEG data with reference to the wavelet transform method were processed and used for comparison. The formula was as follows:

(7) $$W(a,b)=\int_{-\infty }^{\infty }x(t)\psi^{*}(\frac{t-b}{a})dt$$

x(t) is the original signal. $\psi^{*}(\frac{t-b}{a})$ is the wavelet function. *a* is the scaling factor of the wavelet function. *b* is the translation factor. $\psi^{*}$ is the complex conjugate of the wavelet function.

(4) Independent Component Analysis (ICA)

Independent component analysis (ICA) is a commonly used algorithm for blind source signal separation. Its function is to decompose the information of a mixed signal into independent signals [34].

In this process, the ICA model is initialized by the “FastICA” algorithm. Based on fast fixed-point iteration, independent components are separated by maximizing non-Gaussianity. The basic mathematical model of ICA can be expressed as follows. The observation signal matrix *X* is multiplied by the unknown mixing matrix *A* to obtain the independent source signal matrix *S*, as shown in Equation (2):

(8) X=A⋅S

In this model, eight independent components are set for extracting, resulting in the decomposition of eight independent source signals.

(9) $$W^{\left(new\right)}=E\{xg(W^{\left(old\right)}x)\}-E\{g\prime (W^{\left(old\right)}x)\}W^{\left(old\right)}$$

$W^{\left(new\right)}$ is the updated weight matrix used to separate the independent signals in the observation signal X. E{⋅} represents the expectation operation, typically involving averaging over a dataset or probability distribution. $g(W^{\left(old\right)}x)$ refers to the non-linear function g(⋅) applied to the product of the old weight matrix B and the observation signal x. 

To maintain the scalar independence of the independent components, subsequent normalization was performed on the outputs.

(10) $$W={\left(W^{T}W\right)}^{-\frac{1}{2}}W$$

Through the aforementioned processing steps, spatial distribution maps of each independent component were analyzed. They were represented by the column vectors in the mixing matrix *A*, reflecting the projection patterns of the independent components on the scalp.

**Figure 6:**
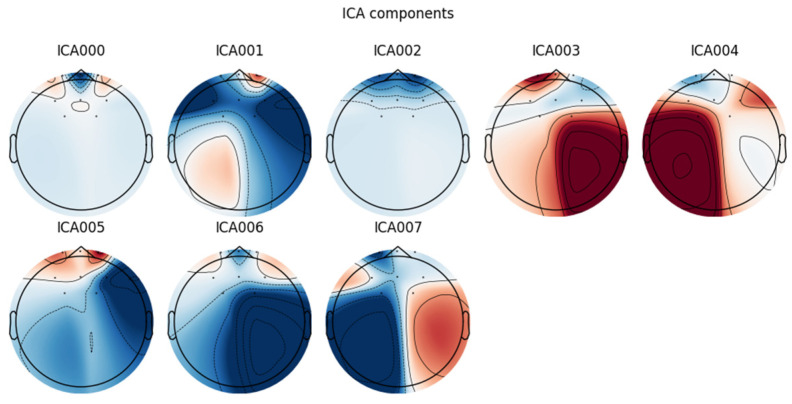
Topographic maps of the 8 independent components obtained from the ICA analysis.

After performing independent component analysis (ICA) fitting, the spatial distributions of each ICA component were visualized as shown in Figure 6. The topographic maps of the eight independent components obtained from the ICA analysis are presented. The projection patterns of each independent component on the scalp are reflected in these topographic maps, providing intuitive evidence for judging whether they represent brain electrical activity or artifacts. For example, the spatial distribution of typical eye movement or electrocardiographic interference is displayed by the components ICA000 and ICA001; hence, they were selected as exclusionary factors.

ICA components related to artifacts were identified and selected for exclusion based on the time series of each component and its topographic distribution. The calculation formula was as follows:

(11) $$X_{corrected}=X-A_{excluded}S_{excluded}$$

$A_{excluded}$ is the excluded mixing matrix, $S_{excluded}$ is the source signal matrix.

(5) Epoch processing

In order to analyze the potential changes associated with specific EEG events, the signal needed to be segmented into segments corresponding to specific events. These segments were classified into datasets representing road hypnosis and normal driving.

In this study, each segment was set as the data from 0.2 s to 1 s after the annotated time point. The positioning of each epoch was at the ERP positions of RH (road hypnosis) and ND (normal driving) in the EEG data. The continuous EEG data were segmented into time periods of equal length. This was achieved by extracting fixed-length data at each epoch position. Taking the EEG data from the 23rd group in the vehicle driving experiment database as an example, the EEG data were divided into 179 epochs. The principle was as follows:

The raw EEG data was denoted as *X*(*t*), where *t* is the time variable. The EEG data were segmented into epochs of length T, corresponding to event i. Therefore, the data for the ith epoch were represented as $X_{i}(t)$, where the range of t is from $t_{1}$ to $t_{2}$.

(12) $$X_{i}(t)=X(t){|}_{t_{1}}^{t_{2}}$$

**Figure 7:**
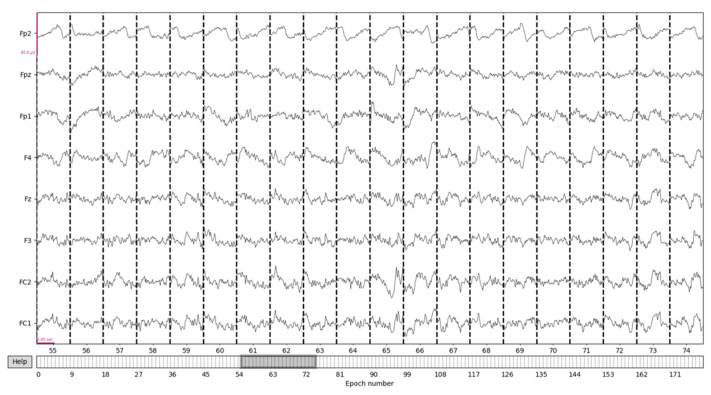
EEG data after epoch processing.

$X(t){|}_{t_{1}}^{t_{2}}$ represents the EEG data extracted within the time range from *t*_1_ to *t*_2_. The average EEG parameters for each channel within the time range from *t*_1_ to *t*_2_ were as follows. EEG data after epoch processing is shown in Figure 7.

(6) Feature extraction

In this step, information from each epoch was subjected to feature extraction and used for PSD analysis. The calculation of PSD was based on Fourier transform. The time–domain signal was transformed into the frequency domain to analyze its frequency components through the calculation of PSD.

The time–domain signal was transformed into the frequency–domain signal through Fourier transform:

(13) $$X(f)=\int_{-\infty }^{\infty }x(t)e^{-j2\pi ft}dt$$

x(t) is the time–domain signal, X(f) is the frequency–domain signal, and f is the frequency.

PSD was obtained by calculating the squared magnitude of the signal’s frequency-domain representation:

(14) $$PSD(f)=\frac{1}{N}{\left|X(f)\right|}^{2}$$

N is the total number of sampling points; X(f) is the Fourier transform of the signal x(t); and ${\left|X(f)\right|}^{2}$ is the squared magnitude of the frequency–domain representation, representing the power at frequency f.

**Figure 8:**
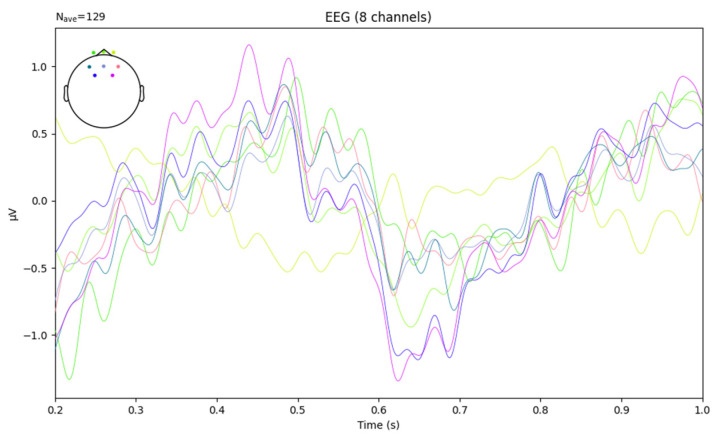
Average EEG parameters for each channel within the time range from *t*_1_ to *t*_2_.

**Figure 9:**
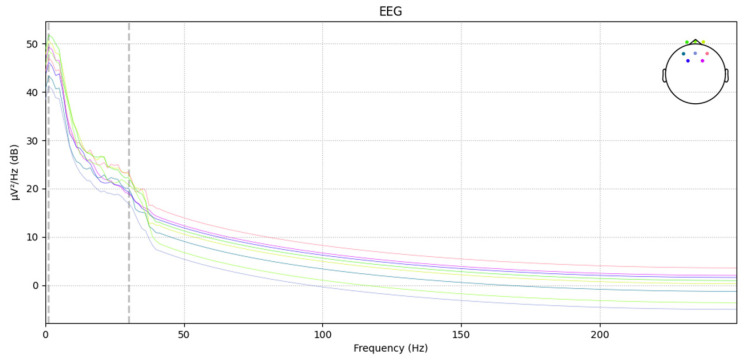
Schematic diagram of EEG data after power spectral density processing.

The average EEG parameters for each channel within the time range from *t*_1_ to *t*_2_ are shown in Figure 8. Schematic diagram of EEG data after power spectral density processing is shown in Figure 9.

After the processing of the EEG data, 7434 data segments were extracted from 41 sets of valid EEG data to construct the road hypnosis vehicle driving experiment database. There were 4327 normal driving segments and 3107 segments characterized by a road hypnosis state. A total of 8623 data segments were extracted from 42 sets of valid EEG data to construct the road hypnosis virtual driving experiment database. There were 3191 normal driving segments and 5432 segments characterized by a road hypnosis state.

### 3.2. Model

**Figure 10:**
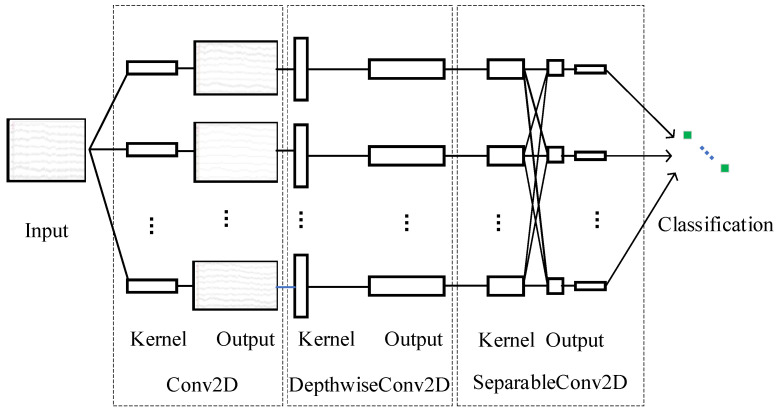
Structure of EEGNet.

Due to the typical temporal characteristics of the experimental data collected, the EEGNet algorithm was selected to establish the road hypnosis state identification model in this study. The structure of EEGNet is shown in Figure 10.

EEGNet consists of three convolutional modules: convolution, depthwise convolution, and pointwise convolution. Parameters of EEGNet are shown in Table 3.

(1) Convolution is a convolutional operation commonly used in convolutional neural networks. Convolution involves multiple convolutional kernels (or filters). The input data are convolved by each convolution kernel, and the convolved results are then summed to produce the output feature map. The local spatial features of the input data can be effectively captured by this type of convolution.

The computation formula is as follows:

Y=X⋅W+b

Y is the output feature. X is the input data. W represents the weight parameters of the convolution kernel. b is the bias term.

(2) Depthwise Convolution

In depthwise convolution, each channel of the input data is convolved separately. The final output feature map is obtained by summing the convolution results across the channels. Through this convolution, deeper features of the input data are more easily extracted.

The computation formula is as follows:

(15) $$Y_{i}=X_{i}\cdot W_{i}i=1,2,\ldots ,C$$

(16) $$Y=\sum_{i=1}^{C}Y_{i}+b$$

$Y_{i}$ represents the output features of the ith channel. $X_{i}$ represents the input data of the ith channel. $W_{i}$ represents the weight parameters of the convolution kernel for the ith channel. b is the bias term. C is the number of input data.

(3) Pointwise Convolution

In pointwise convolution, each pixel of the input data is convolved by a 1×1 convolution kernel. The depth of the input data is reduced to the specified number of output channels.

The computation formula is as follows:

(17) Y=X⊗W+b

*Y* is the output feature. X is the input data. W represents the weight parameters of the convolution kernel. b is the bias term. ⊗ is the pointwise convolution operation.

(4) Batch normalization

In this study, batch normalization was used to accelerate the training process of the model, enhance the stability, and reduce the sensitivity to initialization. Batch normalization was applied to every layer of the network. Data were transformed through the following steps:

The mean of each feature within the given batch of data was calculated as follows:

(18) $$\mu_{B}=\frac{1}{m}\sum_{i=1}^{m}x_{i}$$

$\mu_{B}$ is the mean of batch B. $x_{i}$ is the *i*th data in the batch. m is the batch size.

The variance of each feature within the given batch of data was calculated as follows:

(19) $$\sigma_{B}^{2}=\frac{1}{m}\sum_{i=1}^{m}{\left(x_{i}-\mu_{B}\right)}^{2}$$

$\sigma_{B}^{2}$ is the variance of batch B.

The data were normalized using the calculated mean and variance:

(20) $${\overset{\wedge }{x}}_{i}=\frac{x_{i}-\mu_{B}}{\sqrt{\sigma_{B}^{2}+\varepsilon }}$$

${\overset{\wedge }{x}}_{i}$ is the normalized data point. ε is a small constant used to prevent division by zero.

To enable the batch normalization layer to represent an identity transformation, two learnable parameters, *γ* and *β*, were used to scale and shift the normalized data, respectively.

(21) $$y_{i}=\gamma {\overset{\wedge }{x}}_{i}+\beta$$

$y_{i}$ is the output. 

Preprocessed data and labels from vehicle driving experiments and virtual driving experiments were used for model calibration, training, and validation. To ensure the consistency of data distribution, the hold-out method was used to split the dataset into 60% for the training set, 20% for the validation set, and 20% for the test set. Independent component analysis was performed using the ICA algorithm, and the independent features extracted by the ICA algorithm were input into the EEGNet neural network recognition model to establish the ICA-EEGNet road hypnosis identification model. In order to provide a more objective and multidimensional assessment of the models, road hypnosis identification models were established based on RNN and LSTM using data from vehicle driving experiments and virtual driving experiments, respectively. These models were then compared and validated against the ICA-EEGNet identification model. Time series data were effectively handled by RNN. The sequential information was preserved by the recurrent connections in RNN, enabling the network to capture temporal dependencies and long-range sequential relationships. The num_layers of the RNN value is 2, the input size is 1000, and the hidden size is 128. The gradient vanishing problem of traditional RNN is addressed by LSTM networks through the introduction of “gate” mechanisms. Long sequences and complex temporal features were easily handled by LSTM. The num_layers of the LSTM value is 2, the input size is 1000, and the hidden size is 128.

## 4. Results and Discussion

**Figure 11:**
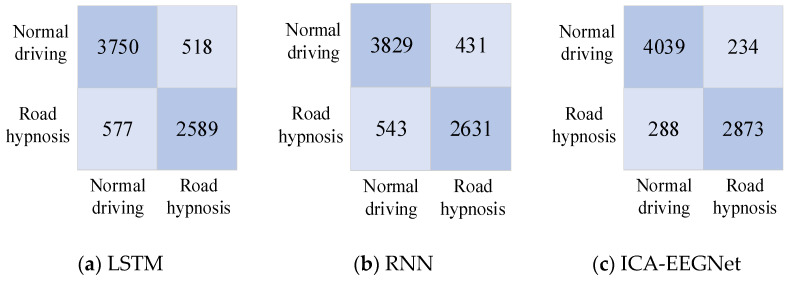
Confusion matrices of virtual driving experiments.

**Figure 12:**
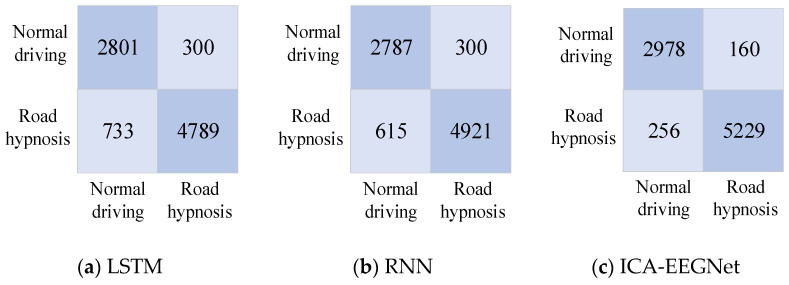
Confusion matrices of vehicle driving experiments.

The confusion matrices for the models obtained after training with LSTM, RNN, and ICA-EEGNet networks are shown in Figure 11 and Figure 12.

The model evaluation metrics utilized in this study included the geometric mean (*GM*), sensitivity (*SEN*), accuracy (*ACC*), and specificity (*SPE*). These metrics are widely used in the fields of machine learning and statistics. They can be employed to evaluate the performance of the ICA-EEGNet, LSTM, and RNN models.

Accuracy refers to the overall proportion of correct predictions made by the model. The calculation formula was as follows:

(22) $$Accuracy=\frac{TP+TN}{FP+FN+TP+TN}\times 100\%$$

Sensitivity (*SEN*) represents the model’s ability to correctly identify positive instances and measures the effectiveness of the model in detecting the road hypnosis state. The calculation formula was as follows:

(23) $$SEN=\frac{TP}{TP+FN}\times 100\%$$

Specificity (*SPE*) represents the capability of negative instances to be correctly identified by the model. The calculation formula was as follows:

(24) $$SPE=\frac{TN}{TP+FP}\times 100\%$$

The geometric mean (*GM*) is an indicator used by the model to assess the balance of predictive performance between positive and negative classes. The calculation formula was as follows:

(25) $$GM=\sqrt{SEN\times SPE}$$

*TP* is the number of true positive samples, *TN* is the number of true negative samples, *FP* is the number of false positive samples, and *FN* is the number of false negative samples.

The accuracy and effectiveness of the prediction results are considered in multiple dimensions. The prediction results of the three models are shown in Figure 12. 

A comprehensive evaluation framework was provided by these evaluation metrics for assessing the overall performance of the models.

**Figure 13:**
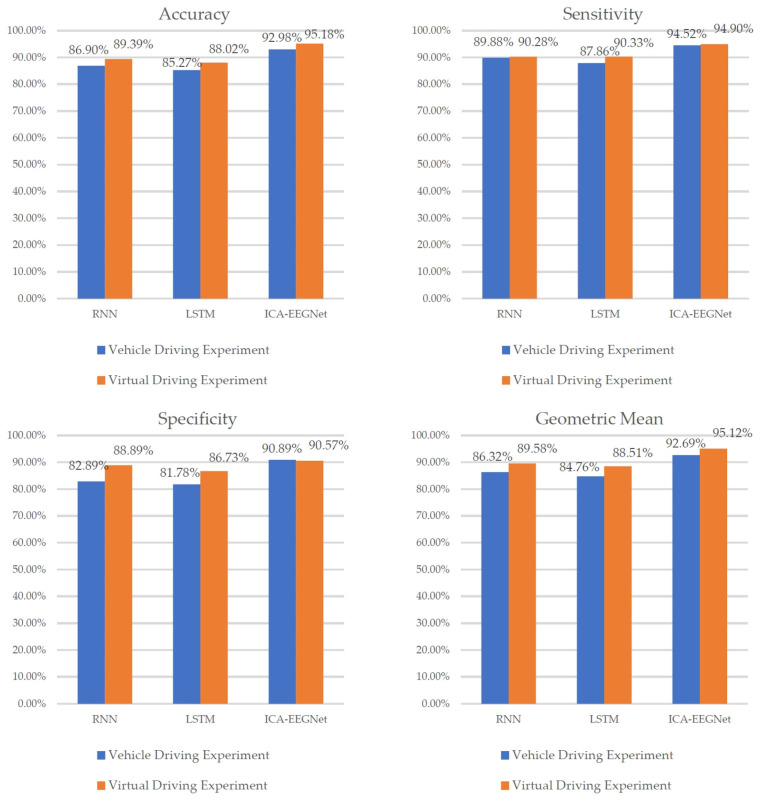
Results of the model evaluation metrics.

As shown in Figure 13, the accuracy, sensitivity, specificity, and geometric mean of EEGNet were all exhibited superiorly to those of RNN and LSTM. This indicates that the road hypnosis state of drivers can be accurately identified from EEG data by the EEGNet neural network. RNN was superior to LSTM in terms of accuracy and specificity. However, there was still a significant gap compared to EEGNet. The overall performance of the RNN model was not stable enough. The lowest identification accuracy and specificity were exhibited by LSTM. The LSTM model was the worst-performing model among the three types.

We found that models established using data from both vehicle driving experiments and virtual driving experiments can exhibit good performance. However, the proportion of road hypnosis occurring in vehicle driving experiments is less compared to virtual driving experiments. This is due to the fact that the road hypnosis state can be more easily induced for drivers in virtual driving experiments. Compared to virtual driving experiments, the data collected from vehicle driving experiments are more representative. However, due to factors such as the larger volume of data from vehicle driving experiments and the varying degrees of road hypnosis induction influenced by the real driving environment, the accuracy of models built based on vehicle driving experiment data is slightly lower than that of models built based on virtual driving experiment data.

In current research, the main methods of analyzing electroencephalogram (EEG) data include entropy analysis, power spectral density (PSD) analysis, and fusion analysis of EEG with other physiological parameters. The uncertainty or randomness of the data is quantified by entropy analysis, which is recognized as an effective means for evaluating the dynamic complexity of a system. However, entropy analysis relies on high-quality data processing, and noise as well as inaccuracies in data collection may lead to misleading analysis results. Additionally, large datasets and complex computational processes are required for entropy analysis, limiting its application in real-time or resource-constrained environments. EEG data is transformed from the time domain to the frequency domain by PSD analysis, providing a detailed breakdown of power distribution across different frequency bands. High-quality features can be provided for models identifying road hypnosis, thereby enhancing the accuracy and reliability of recognition. Therefore, in this study, the PSD analysis method is selected for processing electroencephalogram data.

In this study, the EEG signals are preprocessed through steps and methods including electrode location, re-referencing, filtering, independent component analysis (ICA), and epoch processing. However, this preprocessing method also has some limitations. For example, the quality of the results from independent component analysis (ICA) processing highly depends on the researcher’s judgment and selection, which may introduce subjective bias. In future research, the use of machine learning technologies to train models can be considered to automatically identify artifact components derived from ICA decomposition. This method could enhance the accuracy and automation of artifact recognition, reducing errors caused by subjective factors.

## 5. Conclusions

In this study, the issue of road hypnosis state identification among drivers is addressed by deeply analyzing the drivers’ electroencephalogram (EEG) data. A recognition model based on the ICA-EEGNet network is trained for identifying road hypnosis. The main tasks of this study include:

(1) Vehicle driving experiments and virtual driving experiments are designed and conducted in this study. EEG data are collected from 45 participants during normal driving and road hypnosis. Experts classify the periods of road hypnosis and normal driving by reviewing experimental videos and using scoring methods. A database of road hypnosis is established.
(2) Electrode location, re-referencing, filtering, independent component analysis (ICA), and epoch processing are used to preprocess and extract features from the collected EEG data. The brain features of road hypnosis are effectively extracted with the standardized EEG data. The models for identifying road hypnosis are trained with these features.
(3) EEGNet is utilized to establish a road hypnosis state identification model for vehicle drivers. RNN and LSTM models are introduced for comparison and analysis with the established model. The accuracy, specificity, sensitivity, and geometric mean are used to assess the model. It is indicated from the experimental results that the ICA-EEGNet model has high accuracy and reliability not only in virtual driving experiments, but also in vehicle driving experiments.

In this study, a road hypnosis state identification model based on human EEG data is proposed. The feasibility of using biometric indicators of vehicle drivers to identify road hypnosis is further demonstrated. This provides additional selectable methods and technical support for the real-time and accurate identification of road hypnosis. It holds significant importance for the development of efficient and safe driving assistance systems, potentially enhancing the level of intelligence and active safety of vehicles.

## Figures and Tables

**Table 1:** Basic information of drivers.

Profession	Number of Drivers
Teacher	15
Taxi driver	13
Employees of state-owned enterprises	10
Medical personnel	7

**Table 2:** Examples of raw EEG data.

Parameters	Value
Channels per frame	8
Frames per epoch	1
Events	106,581
Sampling rate (Hz)	500
Reference	unknown
Channel locations	No

**Table 3:** Parameters of EEGNet.

Layer	Parameter	Value
Conv2D	Input Channels	1
	Output Channels	16
	Kernel Size	(1, 51)
	Padding	(0, 25)
BatchNorm2D	Number of Features	16
DepthwiseConv2D	Input Channels	16
	Output Channels	32
	Kernel Size	(1, 10)
	Groups	16
BatchNorm2D	Number of Features	32
AvgPool2D	Kernel Size	(1, 4)

## Data Availability

The data presented in this study are available upon request from the corresponding author. The data are not publicly available due to privacy.
